# Learning to Recognize Phenotype Candidates in the Auto-Immune Literature Using SVM Re-Ranking

**DOI:** 10.1371/journal.pone.0072965

**Published:** 2013-10-14

**Authors:** Nigel Collier, Mai-vu Tran, Hoang-quynh Le, Quang-Thuy Ha, Anika Oellrich, Dietrich Rebholz-Schuhmann

**Affiliations:** 1 European Molecular Biology Laboratory, European Bioinformatics Institute (EMBL-EBI), Wellcome Trust Genome Campus, Cambridge, United Kingdom; 2 National Institute of Informatics, Tokyo, Japan; 3 Knowledge Technology Laboratory, University of Engineering and Technology - VNU, Hanoi, Vietnam; 4 Mouse Informatics Group, Wellcome Trust Sanger Institute, Wellcome Trust Genome Campus, Hinxton, Cambridge, United Kingdom; 5 Department of Computational Linguistics, University of Zurich, Zurich, Switzerland; Indiana University, United States of America

## Abstract

The identification of phenotype descriptions in the scientific literature, case reports and patient records is a rewarding task for bio-medical text mining. Any progress will support knowledge discovery and linkage to other resources. However because of their wide variation a number of challenges still remain in terms of their identification and semantic normalisation before they can be fully exploited for research purposes.

This paper presents novel techniques for identifying potential complex phenotype mentions by exploiting a hybrid model based on machine learning, rules and dictionary matching. A systematic study is made of how to combine sequence labels from these modules as well as the merits of various ontological resources. We evaluated our approach on a subset of Medline abstracts cited by the Online Mendelian Inheritance of Man database related to auto-immune diseases.

Using partial matching the best micro-averaged F-score for phenotypes and five other entity classes was 79.9%. A best performance of 75.3% was achieved for phenotype candidates using all semantics resources. We observed the advantage of using SVM-based learn-to-rank for sequence label combination over maximum entropy and a priority list approach. The results indicate that the identification of simple entity types such as chemicals and genes are robustly supported by single semantic resources, whereas phenotypes require combinations. Altogether we conclude that our approach coped well with the compositional structure of phenotypes in the auto-immune domain.

## Introduction

**Figure 1:**
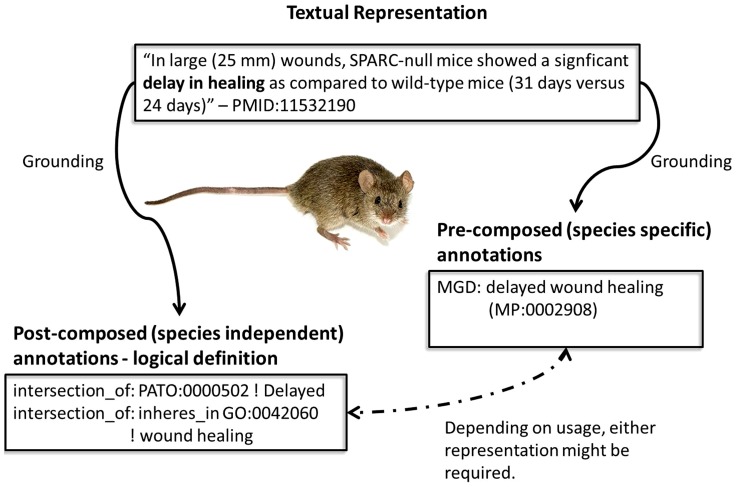
Representation of phenotypes in textual narratives and as pre-composed and post-composed terms. Imagine *Mus musculus* courtesy of George Shuklin published at Wikimedia Commons.

Since the discovery of the relationship between genotype, environment and phenotype, phenotype data has been used to investigate disease–gene relations [1], [2], drug repurposing [3] and in evolutionary studies [4]. A diverse landscape of resources has evolved harboring genotype–phenotype associations such as the Mouse Genome Informatics database (MGD) [5] and the Online Mendelian Inheritance of Man (OMIM) database [6]. This landscape, shown in Figure 1, ranges from narrative descriptions to ontological concepts. Only once we are able to integrate these co-existing data reprentations will be able to fully understand the biological content encoded by each.

While the integration of phenotype data on an ontological level has been demonstrated to enable the prediction of novel gene–disease associations or drug–disease associations [3], the integration of textual data, such as scientific literature, still lags behind. To achieve semantic integration on an ontological level, there was a shift from pre-composed, species-specific phenotype ontologies (e.g. Mammalian Phenotype Ontology (MP) [7]) to a post-composition of phenotype data using species-agnostic ontologies (e.g. Gene Ontology (GO) [8] and PATO [9]). A post-composed phenotype representation requires an entity that is further described based on a quality, e.g. *brown fur colour* or *decreased body weight*. Phenotype data extracted from textual content would have to facilitate both, the normalisation to pre-composed phenotype representations as well as the post-composition of a phenotype.

Furthermore, the data contained within model organism database is obtained through curation of the scientific literature. A need to support database curation work has been identified [10] and current solutions have been found to be insufficient to support the curation workflow [11]. While multiple studies have examined the automatic annotation of genes, proteins and diseases in scientific texts, there is a significant gap in our understanding of how to identify and normalise phenotype mentions. This is partially due to the complexity of the phenotype descriptions, but can also be attributed to incompleteness of phenotype data [12] and a consequent lack of comprehensive semantic resources covering their full scope. Any progress in the automatic identification of phenotypes in the scientific literature would drive the scientific progress in the above mentioned research fields.

This paper presents novel techniques for identifying potential complex phenotype mentions by exploiting a hybrid model based on machine learning, rules and dictionary matching. A systematic study is made of how to combine sequence labels from these modules as well as the merits of various ontological resources such as the Human Phenotype Ontology (HPO) [13], the Foundation Model of Anatomy (FMA) [14] and PATO. We evaluated our approach on a subset of Medline abstracts cited by OMIM for auto-immune diseases. After a review of related research we start start by outlining a conceptual analysis of phenotypes.

## Background

The task of identifying and classifying phenotype mentions in text requires an understanding of the complex nature of their semantics and syntactic structure. In contrast to *simple* entities such as tissues and organs which have a clear structural and spatial basis, the definition and rightful delineation of phenotypes appears puzzling even to researchers and clinicians. This is partly due to phenotypes cutting across both physical objects and processes but also across levels of granularity from the molecular level to the organism. The class of phenotypes is also viewed differently in the clinical and biological data contributing for example to more frequent disease terms in the HPO than in the MP. Phenotypes may be defined experimentally or clinically according to a model reference so that concepts include a notion of *difference to reference model*, leading to a notion of *abnormality* [15]. In the approach that we are taking, we argue that it is vital to annotate surface mentions of phenotypes in a machine readable form that can then be linked to pre-composed phenotype ontologies, and, at the same time, makes explicit their internal dependencies and links their substructures to species-agnostic ontologies to support logical reasoning and hypothesis exploration through post-composition.

The automated recognition of biomedical terms in text has been a highly active area for over two decades and is referred to variously as terminology extraction', term recognition', entity extraction' and named entity recognition' (NER). Most previous NER research has focused on single rather than joint semantic classes such as genes, proteins, cells, anatomical entities and organisms in the experimental biology domain, e.g. [16], and medication, dosage and symptom in the clinical domain, e.g. [17]. Common approaches include supervised machine learning [18]–[21], active learning [22], semi-supervised learning [23], dictionary-based approaches [24], [25], rule-based approaches [26] and hybrid approaches [27]–[30]. Open-source tools for NER include BANNER [21], ABNER [20], LINGPIPE [31], the GENIA tagger [32] and NERSuite, a named entity recognition toolkit based on CRFSuite [33]. Recent community evaluations of state-of-the-art tools for common entity types reported in the BioCreative II [34] and CALBC [35] challenges show quite widely varying F-score performance (see Matching metrics) when trained and tested on the same corpus with the highest scoring approaches generally achieving performance for entity detection and classification of about 80% for genes/gene products, chemicals and diseases and about 90% for organisms. For anatomical entities a granular approach based on 11 levels such as cell, organ and tissue achieved performance of about 71% F-score [36]. In a recent evaluation [37], performance for state-of-the-art NER taggers such as Banner [21], Abner [20] and Lingpipe [31] have been found to offer between 41% and 61% for genes when trained and tested on different corpora. The evaluation in this study was carried out using the partially overlapping annotation method; training was done on standardly available corpora of abstracts such as BioCreative II, JNLPBA [38], GENIA [39] and GeneTag [40] and testing on a newly released full text corpus called CRAFT. We refer readers to the overviews for BioCreative II and CALBC for further background information.

Compared to other entity classes there are very few studies that focus on capturing phenotypes [30], [41]–[43]. Chen and Friedman [41] adapted a rule-based system called BioMedLEE by writing specialised grammatical rules and importing vocabulary from the Unified Medical Language System (UMLS) and the Mammalian Ontology [5]. In a recent study, Khordad *et al.* [42] applied a staged rule-based system on the UMLS, HPO and MetaMap. In our earlier study [30] we provided a comparison of Conditional Random Fields (CRFs), Hidden Markov Models (HMMs) and a hybrid approach against Khordad's method in the domain of human auto-immune diseases. On a two class corpus, performance for phenotypes was 77% F-score for the hybrid system, 65% for the next best performing model CRF, 61% for Khordad's approach and 36% for the HMM. The results indicated the importance of applying a range of resources that can capture phenotypes in experimental papers. [43], Groza *et al.* [44] took a different approach by trying to explicitly model the internal term structure according to qualities and the anatomical entities to which they apply. This is aimed at reducing problems associated with disjoint mentions such as *irregular flared metaphyses… with streaky sclerosis* by normalising to *irregular flared streaky sclerosis metaphyses*. They tested their technique on a corpus of HPO terms under *Abnormality of the skeletal system* (HP:000924).

From these studies we consider the following conclusions to be important: (a) Intuitions about phenotypes are highly variable among experts and therefore good annotation guidelines are necessary for consistency [41], (b) Rule based approaches bootstrapped with ontologies and tools such as the UMLS, HPO and MetaMap are all valuable [41], [42] but their combination with corpus-based approaches can lead to improvements [30], (c) Performance is considered to vary depending on whether phenotypes include both objects and processes [30], [41], (d) Surface term variation remains a key issue [43].

In our approach, rather than solve the problem of identifying free-text phenotypes in one stage, we have divided the task into two stages. (Stage 1) is the identification of candidate terms and, (Stage 2) is candidate confirmation by compositional analysis through grounding to ontologies such as PATO and the FMA, used for the post-composition of phenotype data. The study we report here contributes to both stages of our task, even though Stage 2 is not finished yet. With the work presented in this manuscript, we highlight future directions to be taken in order to enable the identification of the internal structure of phenotypes and their relation to species-agnostic ontologies.

**Figure 2:**
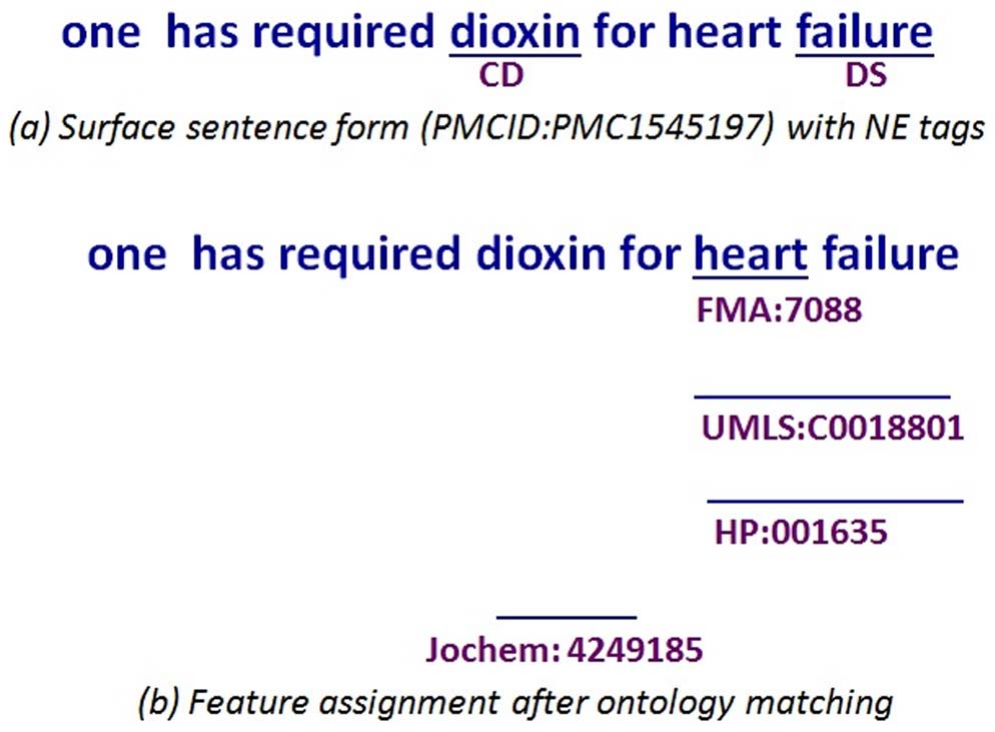
Example tagging of phenotypes along with features from external vocabularies and ontologies.

**Figure 3:**
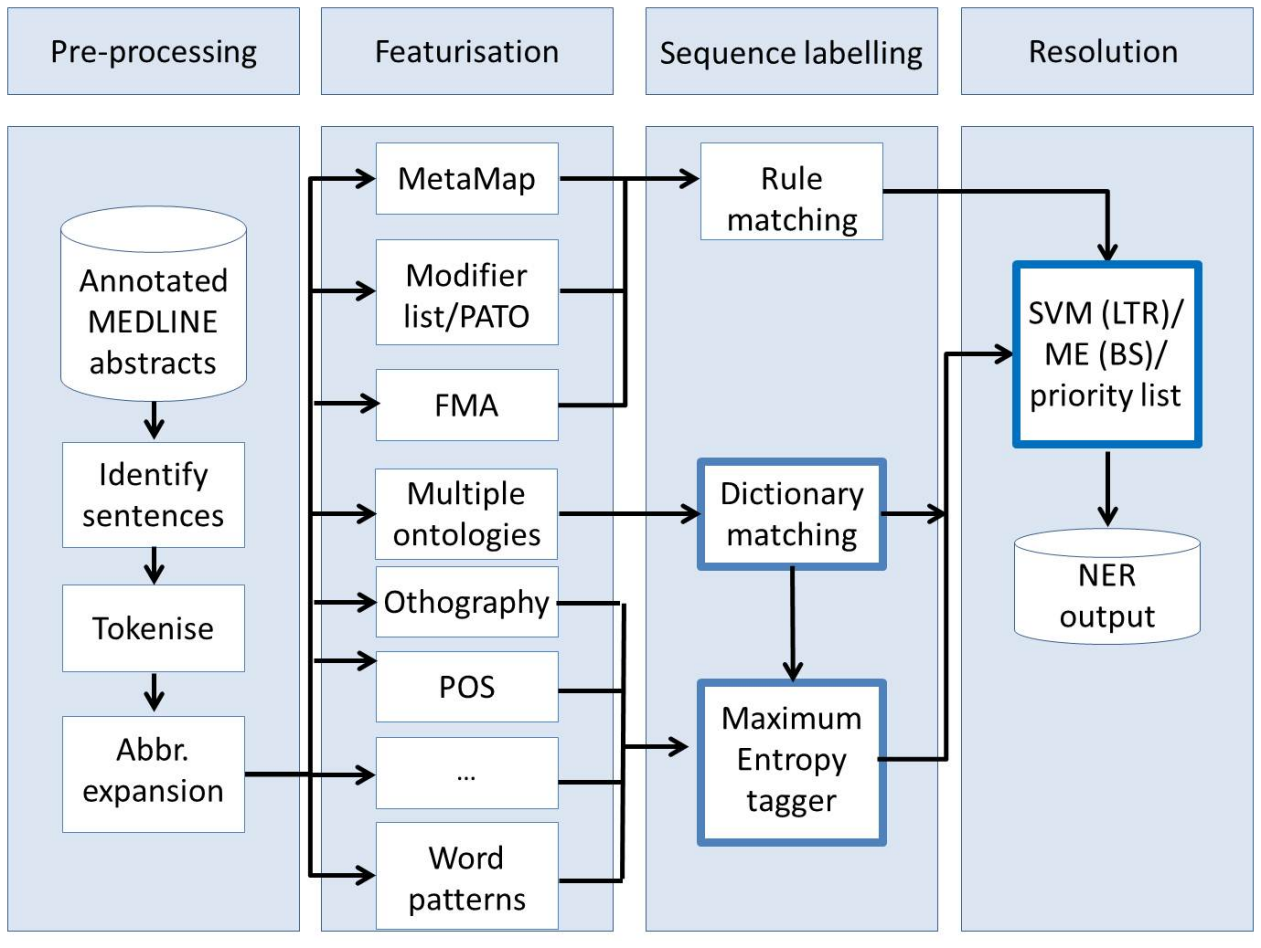
The stages of our experimental phenotype candidate system.

Our previously reported study [30] showed the benefits of a hybrid approach to phenotype candidate recognition. This model combined a state-of-the-art sequence labelling model (Conditional Random Fields) trained on lexical features, with a rule-based MetaMap module and dictionary matching. The target classes were phenotypes and gene/gene products. Hypothesis resolution used a small set of heuristic rules. However, it seemed unlikely that we had reached optimal performance since the domain resources employed and the method we used to combine alternative sequence labeling hypotheses were limited in scope. The study we present here seeks to extend this in a number of important ways:

- We explore additional semantic resources including 320,000 chemical terms from the Joint Chemical Dictionary (Jochem), 9,000,000 gene terms from the National Library of Medicine gene list, 120,000 human anatomy terms from the FMA, 275,000 terms from the UMLS related to diseases and abnormalities, 9,900 phenotype terms from the HPO with 15,800 synonyms, 8,800 phenotype terms from the Mammalian Phenotype Ontology (MP) with 23,700 synonyms, 1400 quality terms from PATO with 2,200 synonyms, species terms from the Linnaeus tool [45] and 5,400 anatomy terms from the Brenda Tissue Ontology [46] with 9,600 synonyms. This is exemplified in Figure 2.
- We evaluate several alternative approaches for hypothesis selection in the merge module by comparing our previous priority list approach to a Maximum Entropy model with beam search (ME+BS) and a Support Vector Machine with learn to rank (SVM+LTR). The full experimental system is illustrated in Figure 3 highlighting the modules where we make our contribution.
- We incorporated four new entity types in our evaluation.

We base our results on the previous study's 122 abstract corpus in order to show a comparison against our earlier methods using phenotype entities.

## Materials and Methods

### Concept analysis

Given the complexity of phenotypes, one important factor we see for achieving automated annotation accuracy is to avoid conceptual inconsistencies in the coding scheme. In this respect principles from formal ontology might be beneficial [47] such as rigorous definition of markable classes as well as semantic linkage to extant standards within the biomedical community. The *de facto* quality assurance standard in NER has been to empirically validate annotation schemes through Cohen's kappa coefficient (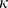) score (e.g. see [48] for a broad discussion). Properly applied this can provide valuable evidence about expert intuition. However if the corpus is not balanced across entity classes then any inferences drawn from agreement on the whole coding scheme becomes weakened. Since it is in practice often difficult to create balanced corpora for NER, if 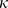 is applied in this way, any changes to systems that improve agreement with the unbalanced corpus may actually move models further away from part of their actual goal which should be to maximise agreement across all classes. Whilst we do not neglect the fact that 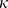 is an important tool for schema development, we also note that empirical studies have pointed to the benefits of formal conceptual analysis techniques such as OntoClean [49]. This is based on an understanding that a failure to clearly define the entities is at least partly responsible for inconsistencies in annotating mentions leading to modeling error.

Here we base our named entities on a formal analysis of biological concepts related to disease by Scheuermann et al. [50] and Beisswanger's BioTop [47]. The entity types we annotate are given in abbreviated upper case form, i.e. GG, CD, AN, PH, DS and OR which we now define.

**Definition: A a gene/gene product (abbreviated as GG) entity is a mention of one of the three major macromolecules DNA, RNA or protein.**

Examples include: [cryoglobulins], [anticariolipin antibodies], [AFM044xg3], [chromosome 17q], [CC16 protein].

**Definition: A chemical or drug entity (abbreviated as CD) entity is a mention of a chemical part or family other than genes and gene products (DNA, RNA and protein).**

Kim *et al.* [51] indicate in the GENIA encoding manual that chemical entities contain element chemicals and compound chemicals, where the later can be either organic or inorganic. Here we apply a granular cut off to organic chemicals, considering that proteins and nucleic acid compounds are a separate entity class called GG. Small biomolecules are included within the scope of CD.

Following Corbett et al. [52] and the CALBC challenge guidelines [53] we include chemical compounds, molecular formulas, IUPAC nomenclature and drug names within scope.

Examples include: [Panadol], [antibiotic], [calcium], [3-ethyl-2-methylhexane] and [C_6_H_12_O_6_].

**Definition: An anatomy entity (abbreviated as AN) is a mention of an anatomical structure or other physical component within or on the surface of the human or mouse body, including organs, cells, portions of bodily substances such as blood, body fluids, tissues and their combinations.**

The definition here follows on from that in Scheuermann *et al.* [50] except that (a) we apply a granular cut off at the level of cell (but include cell internal structures such as nucleus). Units smaller than a cell may be included in either CD (chemical or drug) or GG (gene/gene product), and (b) we apply AN only to the morphology of human and mouse organisms.

Examples include: [endothelial cells], [liver], [nervous system], [HeLa cells], [left collar bone], [both kidneys].

**Definition: A phenotype entity (abbreviated as PH) is a textual mention that describes an observable and measurable characteristic of an organism. Phenotype entities can be further broken down into an affected entity and a describing quality for that entity.**

Examples include: [differences in the levels of the protein], [airway inflammation], [absent ankle reflexes].

Our definition of phenotype require two caveats (a) in contrast to Khordad *et al.* [42] we did not apply a granular cut off at the level of cell, and (b) because of the diversity of phenotypes across organisms we took a decision to focus our definition of this entity on mouse as a model organism and human as the most important species. Following the discussion of phenotypes as processes in physiology [3] we include some mentions of processes within the scope of our annotation schema.

**Definition: A disease entity (abbreviated as DS) is a mention of a disposition to undergo pathological processes in an organism because of one or more disorders in that organism.**

Examples include: [Felty's syndrome], [rheumatoid arthritis], [heterozygous C2 deficiency], [Paget's disease], [inherited skeletal dysplasia].

**Definition: An organism entity (abbreviated as OR) is mention of a type of living biological system which functions as a stable whole.**

This definition is adapted from Beisswanger *et al.*'s [47] concept for *living organism* (BioTop ID LivingOrganism). In common with both BioTop and the GENIA ontology [54] we include both multi-cellular and mono-cellular organisms within this definition. For simplicity we also include viruses within this definition.

Our definition of an organism entity encompasses both mentions of names of species as well as individuals of those species. Individuals can be named or in some cases described.

Examples include: [Hepatitis type B virus], [food sanitation inspectors], [cholera cases], [hypergammaglobulinaemic patients], [45-year-old male], [asthmatics].

We should not however ignore the important lexical and syntactic considerations about how to annotate mentions in text. Within the annotation guidelines we developed we further describe whether specific, generic, underspecified and negatively quantified mentions qualify. This is summarised in Table 1. We follow [55] in differentiating between (a) *specific mentions* with specific reference to objects or group of objects, (b) *generic mentions* which refer to the kind of entity, (c) *underspecified mentions* which have non-generic non-specific reference, e.g. everyone, and (d) *negative mentions* which refer to the empty set of the kind of entity.

**Table 1:** Referential semantics and scoping of mentions by entity type.

	specific	generic	underspecified	modifiers	conjunctions	processes	negation
	reference	reference	reference		disjunction		
GG	Yes	Yes	No	No	Yes^1^	No	No
DS	Yes	Yes	No	No^4^	Yes^1^	No	No
CD	Yes	Yes	No	No	Yes^1^	No	No
OR	Yes^2^	Yes	No	No	Yes^1^	No	No
AN	Yes	Yes	No	Yes^3^	Yes^1^	No	No
PH	Yes	Yes	No	Yes^3^^,^ 7	Yes^1^	Yes^5^	Yes^6^

Notes on annotation:

1 Where there is elision of the head, e.g. [IA/H5 virus], then annotate the whole expression. Otherwise annotate each expression separately, e.g. [IA virus] and [H5 virus].

2 Markable expressions include specific people, e.g. [Jane] as well as definite noun phrases such as, the [24-year-old man].

3 Quantitive modifers are included, e.g. [both kidneys] as well as spatial modifiers, e.g. [left collar bone].

4 When modifiers are considered to be part of the disease name they are included, e.g. [highly pathogenic avian influenza], [end-stage renal disease].

5 We exclude however finite verb forms, infinite verb forms with to', verbs in a progressive or perfect aspect, verb phrases, clauses or sentences and any phrase with a relative clause or complement clause.

6 If the negation appears in a noun phrase with an anatomical entity then we generally allow it, e.g. [absent ankle reflexes], [no left kidney].

7 Qualitative modifiers are included. For example, physical components: [black hair], underspecified ranges: [normal height], locational modifers: [low set ears], and level modifiers: [quite small fingers].

### Data preparation

The Phenominer A corpus (available as Data S1 or on request from the first author) contains 122 abstracts selected from Medline. 19 auto-immune diseases were selected from OMIM and from these records citations were then chosen. Citations were only selected for the corpus if they contained the auto-immune disease term and at least one term from either OMIM's clinical synopsis field, the HPO [56] or the MP [57]. This strategy is designed to ensure that the abstracts have some association to phenotypes or anatomical entities in addition to the disease itself. Table 2 shows the 19 diseases and the corresponding affected organism. Descriptive statistics are shown in Table 3. Despite being small, the number of annotated entities is consistent with several previous specialised studies, e.g. [18], [42], [58].

**Table 2:** Auto-immune diseases from OMIM represented in the Phenominer A corpus.

Disease	Organism
Auto immune thyroid disease	human
Auto immune skin diseases	human
Immune mediated diseases	human
Immuno-mediated gastrointestinal diseases	human
Celiac's disease/Caliac disease	human
Grave's disease/Grave disease	human
Hashimoto's disease/Hashimoto disease	human
Crohn's disease/Crohn disease	human
Addison's disease/Addison disease	human
Type 1 diabetes	human
Rhematoid arthritis	human
Multiple sclerosis	human
Systemic lupus erythematosus	human
Asthma	human
Familial psoriasis	human
Auto immune encephalomyeliti	mouse
Inflammatory arthritis	mouse
Histamine sensitization	mouse
Mouse lupus	mouse

**Table 3:** Descriptive statistics for entities in the Phenominer A corpus.

Entity	# Entities	# Unique Entities	Average length of entity
PH	472	393	3.0
OR	764	402	1.8
DS	875	270	1.9
GG	1611	885	1.7
AN	188	132	2.2
CD	48	31	1.4

Corpus annotation was carried out by a single experienced annotator who had previously worked on the GENIA corpus and the BioNLP shared task corpus. The annotator is not one of the authors and is independent from the experiments. Tool support was provided by the BRAT annotation tool (http://brat.nlplab.org). Entities were annotated using the commonly used **B**egin **I**n **O**ut annotation scheme, so for example *between airway responsiveness* would be annotated with the sequence *O B-PH I-PH* where ‘O’ denotes a word outside an entity, ‘B’ a word at the beginning of an entity, and ‘I’ as a word inside an entity.

### Experimental system

Our experiments were divided into two stages. In the first stage we wanted to find the optimal combination of external resources for the range of entity types described above. The hypothesis resolution approach used in these experiments was the same as our previous method in [30], i.e. a priority list. After this we froze the external resource features and proceeded to compare hypothesis resolution strategies. Three approaches were evaluated.

Figure 3 shows the complete system. The pre-processing stage collects the abstracts from the source provider (PubMed), splits the text into sentences and tokenises using the OpenNLP library with a Maximum Entropy model. This is then followed by abbreviation expansion using BioText [59]. Abbreviations are replaced using their full forms if they are given in the abstracts.

Three distinctive classification modules are applied within the NER system. The first of these Rule matching' follows a similar approach to Khordad's use of MetaMap (UMLS) with staged rules for post-processing [42] and a modifier list derived from HPO (85 terms) and PATO. We also added the Gene dictionary from NCBI to this module in line with our original experimental system. The second module is Dictionary matching'. This uses a longest string matching approach to identify term candidates for each entity class in the relevant ontology. For example, FMA and the Brenda tissue ontology for AN entities, Jochem for CD entities, PATO/MP/HPO for PH entities and so on. A precise list of the resources and term counts is given in the Introduction. Finally the third module is a Maximum Entropy with Beam Search (ME+BS) supervised sequence labeler using multiple linguistic features associated with the training corpus. Features include the focus word, surrounding context words, part of speech labels. Additionally we added semantics tags from a ME+BS model trained on the JNLPBA corpus [38] and Linnaeus [45]. The JNLPBA corpus contains 2000 Medline abstracts selected by a search using terms *human, blood cell, transcription factor* and then hand annotated for 5 NE classes including RNA, DNA and protein which we merge to form our GG class.

#### Experiment 1: Rule-based hypothesis resolution with multiple ontologies

Based on our best performing approach from [30] we applied a hybrid method to entity recognition across the six classes. For the variable component we wanted to test the influence of each standard ontology and so used ablation to knock out' each resource in turn, thereby measuring its contribution to the accuracy for each class.

A Maximum Entropy model with Beam search (ME+BS) [60] was selected as the machine learning method using the Java-based OpenNLP toolkit (http://opennlp.apache.org/) with default parameters. At this stage we treat NER assignment of tags as a sequence labeling problem. This is implemented through a sliding window of features around the target word being classified and by optimisation of the sequence of tag assignments during the decoding phase, i.e. through the beam search algorithm.

The Resolution module for deciding the final class of the entity based on competing hypotheses used a ranked priority list of hand built rules as described in our previous experiments. In summary this gives priority to labels in the following order: 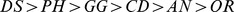. This judgment was based on introspective analysis of terms, e.g. that phenotypes usually contain an anatomy or a gene component (*pannus formation*, *elevated serum levels of cartilage oliomeric matrix protein*), and that genes sometimes contain a organism name (*mouse H19 gene*, *mouse ABcg2/Breast cancer resistance protein (BCRP) gene*). However organism names never contain a gene name and anatomy names.

In contrast to straightforward supervised learning our system combines the traditional machine learning based approach to NER, with its advantage of context sensitivity and compensation for lexical variation, with other approaches that bootstrap extant domain vocabularies. For example, the Mammalian Phenotype Ontology contains the term *skull anomaly, congenital* but in the text this may appear as the more general mention *congenital anomalies*. A number of string matching algorithms have been adapted for identifying synonyms and related terms such as [25] whilst others have tried to normalise external resources to a standard format [61]. As we might expect, performance has been found to vary considerably across resources and entity types. Here we use a simple longest string matching strategy between the text and the term in the external resource but normalising for plurals. As noted previously, hypothesis resolution is conducted sentence by sentence using a staged set of rules, given here from [30]:

1. We combine the putative entity labels by collecting any entity-specific result that has been proposed by at least one module. This is intended to maximise recall. The O tag (non-entity label) has the least priority.
2. Based on our ontological analysis of PH and GG it is often possible for a GG to form a fully embedded part of a PH mention. For example, [high [IgE]*_GG_* levels]*_PH_*. We therefore apply a longest span rule and give priority to PH over GG giving [high IgE levels]*_PH_*.
3. If there is a boundary conflict, we merge neighbouring entity mentions that share parts of their token sequence. For example, if we have [AB]*_GG_* and [BC]*_PH_* then we merge them into one phrase [ABC] and label it with the highest priority tag, i.e. PH. Although this appears rare in GG and PH we included this rule for expandability when we want to introduce further entity classes.

The testing framework was 10-fold cross validation using the Phenominer A corpus described in Data Preparation, i.e. the corpus is partitioned in 10 rounds so that 9 equal parts are used for training the models and the remaining 1 equal part of unseen data is used for testing. Results are collected from each of the 10 testing partitions and the accuracy is calculated against the reference standard.

Our primary purpose in these experiments is to focus on the contribution made for phenotype candidate recognition but at the same time to take into consideration the effects that resources have on the recognition performance of other entity types.

#### Experiment 2: Alternative hypothesis resolution strategies

**Figure 4:**
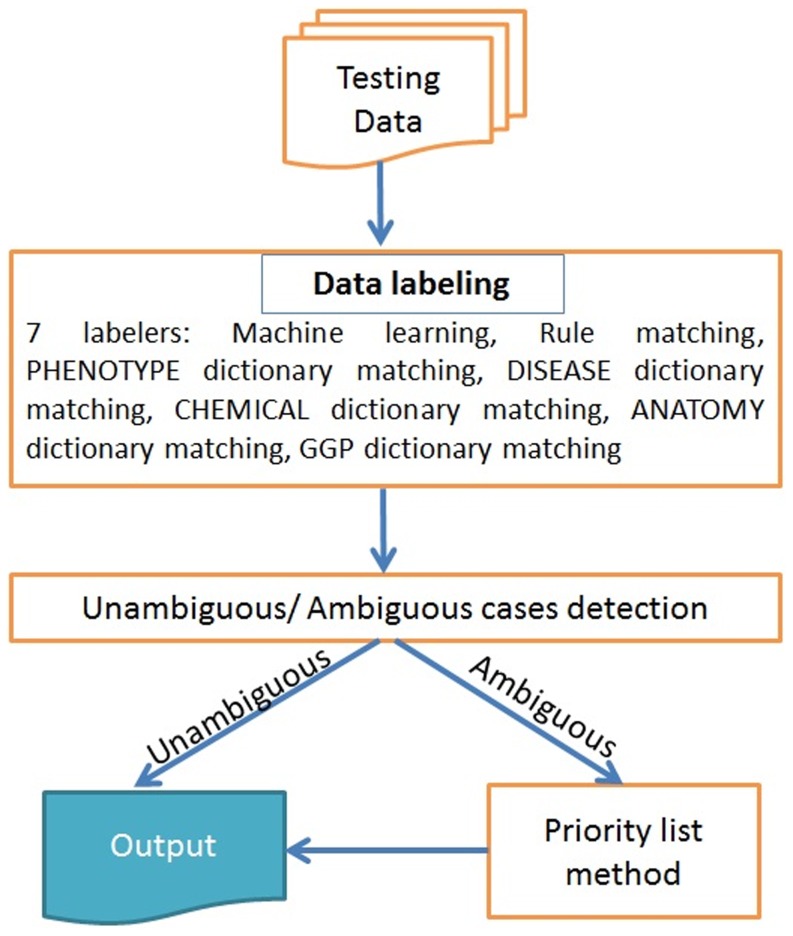
Hypothesis resolution using a priority list.

The baseline method we chose used the priority list approach used in Experiment 1. This is shown as a flow diagram in Figure 4. We have outputs from 7 labelers: Rule matching, PH dictionary matching, DS dictionary matching, CD dictionary matching, AN dictionary matching and GG dictionary matching and a ME+BS tagger. Outputs from these labelers were screened using an Unambiguous/Ambiguous case detection module. Where we detected a labeling conflict, i.e. an ambiguous case, we used the priority list approach to resolve this and chose only one output, otherwise, the agreed output was considered as the final output.

**Figure 5:**
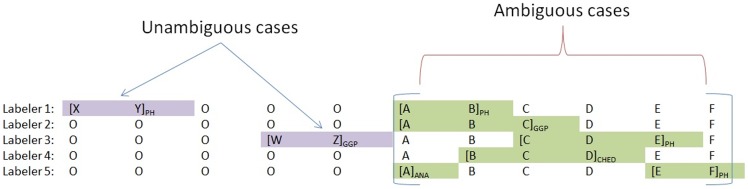
Handling ambiguous versus unambiguous cases.

Figure 5 illustrates a possible scenario for an unambiguous and an ambiguous case. Labelers 1 to 5 represent the different modules providing alternative hypotheses. In the unambiguous case two label sequences are proposed as PH for *X Y* and GG for *W Z* but there is no conflict and the final labelling will be *B-BF I-BF O B-GG I-GG* under the BIO scheme. In the ambiguous case there are multiple alternative hypotheses suggested for the first token *A* with the labelers suggesting PH, GG, O and AN.

Whilst our priority list approach seemed to perform adequately we wanted to investigate other hypothesis resolution strategies based on machine learning using the 10-fold validation framework we employed in Experiment 1.

#### Maximum entropy model with beam search

**Figure 6:**
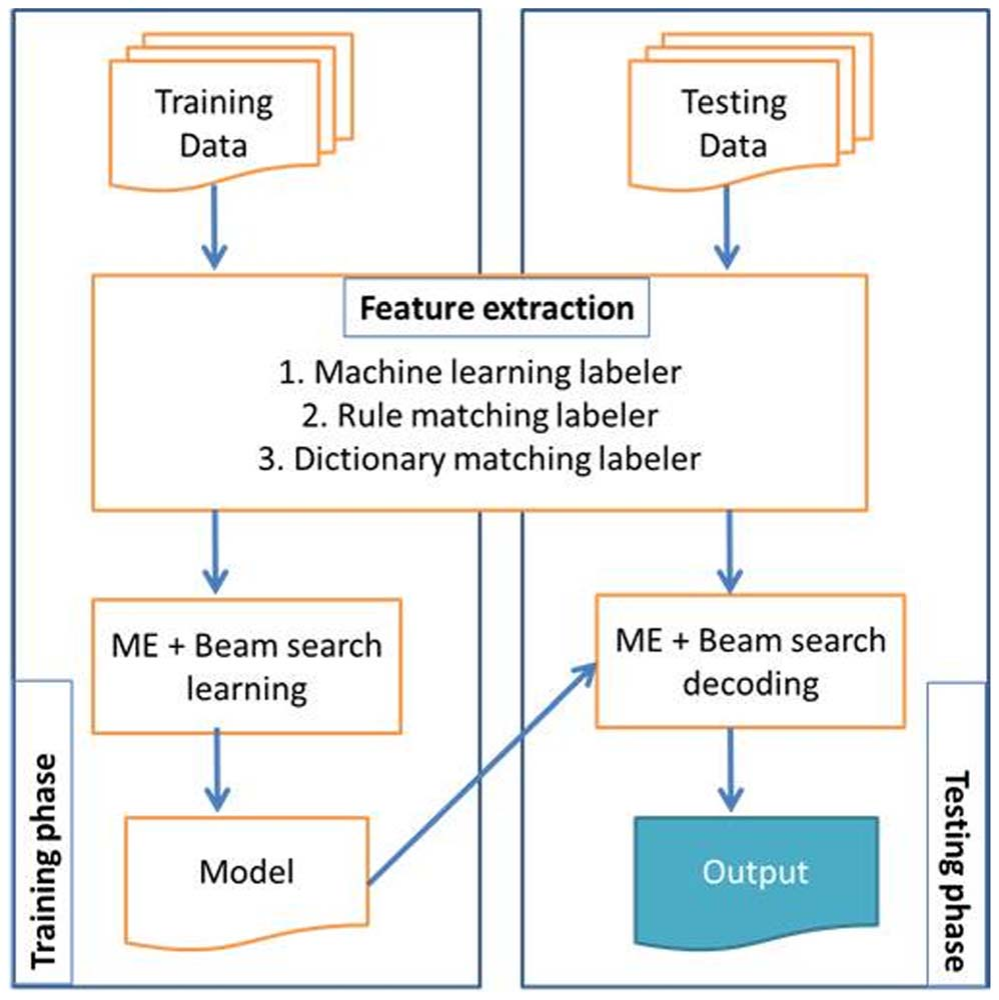
Hypothesis resolution using maximum entropy with beam search (MS+BS).

The first alternative that we explored was a Maximum Entropy Model [60], [62] with beam search (ME+BS) as shown in Figure 6. The maximum entropy estimate is the least biased estimate possible on the given information, i.e. it is maximally noncommittal with regard to missing information.

The original Maximum Entropy model for named entity labeling used the Viterbi algorithm for decoding, a dynamic programming technique. Instead of Viterbi we used beam search decoding. Beam search is a variant of breadth first search using a parameter 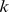 to decrease the search space (in our model, we set 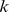 = 3). The advantage of using beam search is that it allows the tractable use of maximum entropy for each labeling decision but forgoes the ability to find the optimal label sequence using dynamic programming techniques. The computational complexity of beam search decoding is 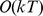 compare to 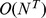 for Viterbi decoding (in which, 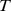 is the number of words, 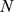 is the number of labels). To implement ME+BS, we used the Java-based OpenNLP toolkit (http://opennlp.apache.org/) with default parameters.

The outputs from the machine learning (ME+BS) labeler, rule based labeler and dictionary based labelers were used as features to train the ME+BS resolution model, then, we used this model to choose the final output. Note that in contrast to the other two hypothesis resolution methods, this approach did not apply screening for unambiguous or ambiguous cases since it resolved the conflict with the sequence labeling technique. The features we employed are shown in Table 4.

**Table 4:** Features used by the Maximum Entropy model for hypothesis resolution.

No.	Feature	Example
1	Current word	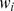
2	Context words	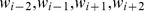
3	ME+BS labels	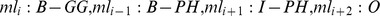
4	Rule matching labels	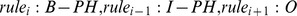
5	PH dictionary labels	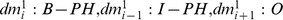
6	DS dictionary labels	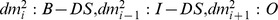
7	CD dictionary labels	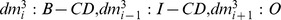
8	AN dictionary labels	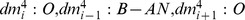
9	GG dictionary labels	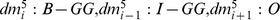

#### Support vector machine with learn-to-rank

**Figure 7:**
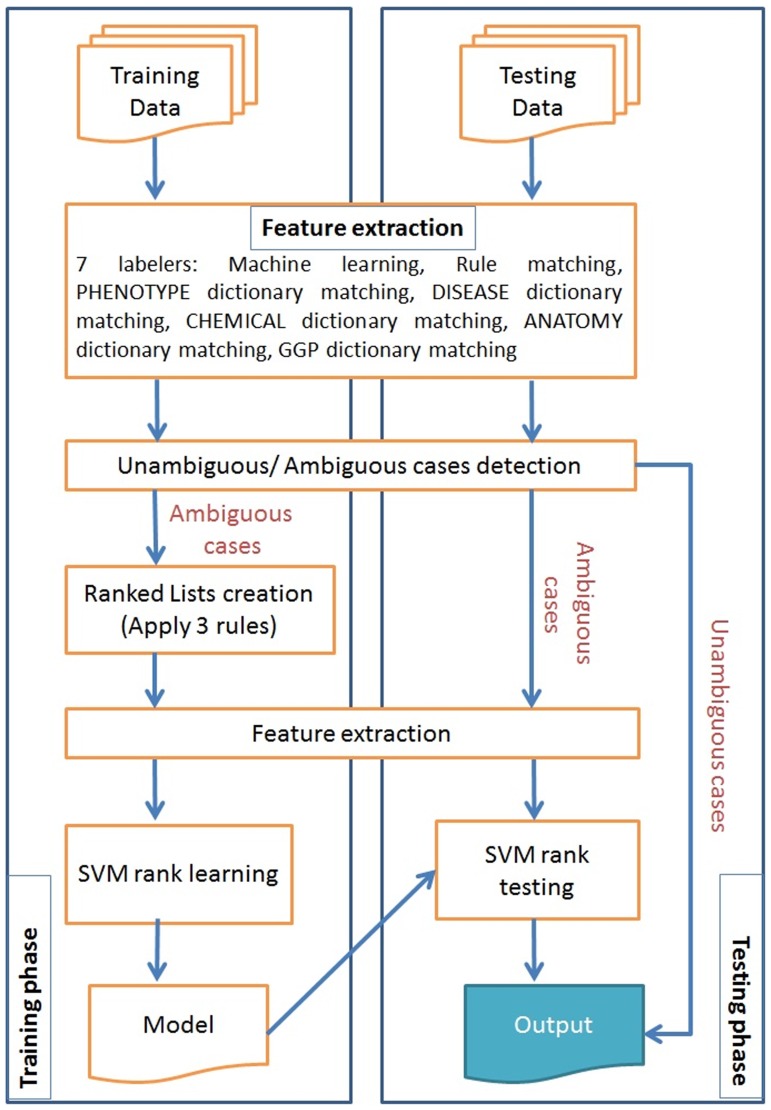
Hypothesis resolution using support vector machine and learn to rank (SVM+LTR).

With an appropriate scoring function it is possible to consider the choice of alternative named entity labels from the various modules and dictionaries as a ranking problem. This means that each source is scored against certain criteria and the scores are then compared with the highest one being chosen. We implemented this using the SVM*^rank^* software from Thorsten Joachims at Cornell University (http://www.cs.cornell.edu/people/tj/svm_light/svm_rank.html). The experimental system is shown in Figure 7. Essentially processing proceeds token by token through the sentence. When an ambiguous token is discovered - one in which there is more than one alternative label being proposed by the labelers - SVM*^rank^* is used to decide on the named entity tag.

In the first stage we applied the same screening technique as the priority list approach for unambiguous/ambiguous case detection. Unambiguous cases are considered as the final output labels with no further processing. For ambiguous cases, three rules were used to create the ranked lists. Through the feature extraction module, these ranked lists were used to trained an SVM learn-to-rank model. Then we used this model to choose the final output if conflict appeared in the test set.

In training ranking was decided by the following three heuristic rules:

1. Candidates having the same label with the training annotation receive the highest rank. Among these, candidates matching closer to the left hand side of the annotated sequence have a higher rank than candidates which match further to the right since we process the sequence in a left to right order.
2. Candidates having a partial overlap in tag assignment with the training annotation receive the second rank. Among these, candidates matching closer to the left hand side of the sequence have a higher rank than candidates which match further to the right. Again this is because we process the sequence in a left to right order.
3. Candidates that have no overlap in tag assignment with the training annotation receive the lowest rank.

SVM*^rank^* is trained using these heuristics and compared against the ME+BS and priority list methods.

### Matching metrics

We follow standard metrics of evaluation for the task using F1, i.e. the harmonic mean of recall and precision. This is calculated as follows:
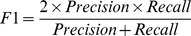
(1)where precision indicates the percentage of system positives that are true instances, and recall indicates the percentage of true instances that the system has retrieved. More formally this is shown by the following two equations and Table 5.
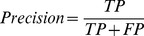
(2)

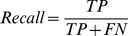
(3)


**Table 5:** Defining the test metrics.

		Gold standard class	
		True	False
System	True	TP	FP
			(Type 1 error)
class	False	FN	TN
		(Type 2 error)	

Different applications require a different approach to defining a true positive. In these experiments we consider a correct match to be recorded when a *partial matching* occurs, i.e. when the span of text that is manually annotated in the gold standard corpus and the span of text output as an entity by the NER tagger partially overlap. For example a system annotation of *[median cleft lip]/palate* would be judged correct for a gold standard annotation of *median [cleft lip/palate]*. Various authors in the biomedical NER domain such as [63] have offered a reason for why this or other methods such as sloppy left boundary matching might be preferred to strict matching for genes and proteins. In summary it is thought that with partial matching, for the entity types examined so far, the core part of the entity was in most cases correctly found. In contrast, strict matching places too much faith on possibly arbitrary annotation choices as well as corpus selection, meaning that system performance might not be repeated on new texts outside the narrow domain of the gold standard. However whilst our focus is on partial matching we have included results for exact matching for comparison purposes.

### Significance tests

Based on [64], [65], we compared performance across different systems using an approximate randomization approach for testing significance. In order to calculate significance for two different systems (system A and system B) on the Phenominer corpus (with 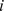 sentences), we performed the following steps:

- (1) Compute micro-average F-scores using 10 fold cross validation from each system and note the difference in performance 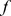 = 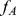
−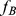;
- (2) Generate set 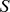 (with 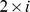 sentences) by taking the outputs from the 10 fold validations on the two systems;
- (3) Obtain 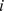 sentences randomly from set 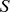 to create set 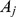, the remainder of 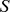 is set 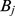 (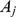 is used for system A and 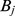 is used for system B);
- (4) Calculate 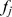 = 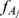
−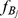 (in which, 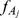 and 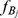 are micro-average F-scores using 10 fold cross validation for set 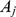 and 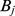 respectively).

Steps 2–4 were repeated 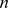 times (we set 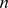 = 1000 as in [64]). The number of times that 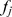
−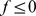 in 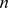 loops divided by 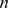 is the p-value between system A and system B.

## Results and Discussion

### Results

#### Resources contribution

Table 6 shows the contribution by each external resource by comparing F-scores for each NE class when it is removed from the system. As noted above, a partial matching metric was used. For comparison we include the same evaluation using exact matching in Table 7. Performance for PH is notably lower using exact matching, indicating the challenge caused by their high variability and length (see Table 3). The last row is the result when applying all resources; the hypothesis resolution module used the priority list method. All external resources help to increase the F1, but the contribution varies among them. Some resources help to increase the result greatly whereas others just bring minor improvements; some resources seem to be important for only one NE class but others affect many entities.

**Table 6:** Performance of named entity recognition using using partial matching for ME+BS in machine learning labeler and priority list in resolution module.

External resources										*Named entity classes*						
J	U	H	M	G	L	F	P	C	B	*PH*	*OR*	*AN*	*GG*	*CD*	*DS*	*ALL*
−	+	+	+	+	+	+	+	+	+	73.7	75.6	76.2	**71.0**	78.9	74.2	68.8
+	−	+	+	+	+	+	+	+	+	**68.3**	72.1	76.8	83.2	78.7	**61.4**	73.1
+	+	−	+	+	+	+	+	+	+	**61.8**	74.0	77.1	84.8	80.4	73.6	73.7
+	+	+	−	+	+	+	+	+	+	**54.4**	75.2	75.6	85.0	80.4	73.2	72.1
+	+	+	+	−	+	+	+	+	+	74.6	75.4	77.1	**82.7**	80.4	74.3	78.9
+	+	+	+	+	−	+	+	+	+	73.2	**49.9**	76.7	85.2	79.3	73.8	77.4
+	+	+	+	+	+	−	+	+	+	74.9	75.4	**59.0**	85.2	80.4	74.3	77.1
+	+	+	+	+	+	+	−	+	+	**74.7**	75.4	77.1	85.2	80.4	74.3	79.1
+	+	+	+	+	+	+	+	−	+	74.9	75.4	77.1	85.2	**41.6**	74.3	75.2
+	+	+	+	+	+	+	+	+	−	74.9	75.4	**76.0**	85.2	80.4	74.3	79.1
+	+	+	+	+	+	+	+	+	+	74.9	75.4	77.1	85.2	80.4	74.3	79.2

Each horizontal row shows a combination of features and the associated F-scores for each class on test data. *ALL* shows micro-averaged F-score. Key to external resources: J: JNLPBA model, U: UMLS and MetaMap, H: Human Phenotype Ontology, M: Mammalian Phenotype Ontology, G: Gene Dictionary from NCBI, L: Linnaeus, F: Foundation Model of Anatomy, P: Phenotypic Trait Ontology, C: Jochem's dictionary, B: Brenda Tissue Ontology.

**Table 7:** Performance of named entity recognition using exact matching for ME+BS in machine learning labeler and priority list in resolution module.

External resources										*Named entity classes*						
J	U	H	M	G	L	F	P	C	B	*PH*	*OR*	*AN*	*GG*	*CD*	*DS*	*ALL*
−	+	+	+	+	+	+	+	+	+	36.0	61.3	58.0	48.5	71.3	55.3	50.1
+	−	+	+	+	+	+	+	+	+	35.3	60.4	58.0	57.1	71.2	49.4	52.2
+	+	−	+	+	+	+	+	+	+	33.5	58.2	58.0	56.4	71.3	54.3	53.0
+	+	+	−	+	+	+	+	+	+	30.0	57.4	57.4	58.7	71.3	53.7	52.7
+	+	+	+	−	+	+	+	+	+	36.0	61.3	58.0	58.2	71.3	55.3	54.4
+	+	+	+	+	−	+	+	+	+	35.4	35.6	57.6	59.2	70.8	55.0	53.2
+	+	+	+	+	+	−	+	+	+	36.3	61.3	39.2	59.2	71.3	55.3	54.5
+	+	+	+	+	+	+	−	+	+	35.5	61.3	58.0	59.2	71.3	55.3	55.4
+	+	+	+	+	+	+	+	−	+	36.3	61.3	58.0	59.2	38.4	55.3	55.3
+	+	+	+	+	+	+	+	+	−	36.3	61.3	56.9	59.2	71.3	55.3	55.3
+	+	+	+	+	+	+	+	+	+	36.3	61.3	58.0	59.2	71.3	55.3	55.4

Each horizontal row shows a combination of features and the associated F-scores for each class on test data. *ALL* shows micro-averaged F-score. Key to external resources: J: JNLPBA model, U: UMLS and MetaMap, H: Human Phenotype Ontology, M: Mammalian Phenotype Ontology, G: Gene Dictionary from NCBI, L: Linnaeus, F: Foundation Model of Anatomy, P: Phenotypic Trait Ontology, C: Jochem's dictionary, B: Brenda Tissue Ontology.

Using the ME+BS model trained on the JNLPBA corpus brings much better results for GG (85.2% compared to 71.0%) whereas using Gene Dictionary from NCBI helped GG to gain from 82.7% to 85.2%. Both the HPO as well as the MP help PH to increase from 61.8% and 54.4% to 74.9% respectively. The use of PATO allows the PH score to increase only slightly (from 74.7% to 74.9%). Linnaeus seems to play an important role in recognizing OR; when removing Linnaeus, OR's result is down significantly from 75.4% to 49.9%. Similarly, removing the FMA results in a drop in performance for AN from 77.1% to 59.0%, but removing the Brenda Tissue Ontology just makes AN's result drop slightly to 76.0%. Jochem's dictionary focuses on CD, resulting in a very large increase of 38.8% (from 41.6% to 80.4%). Using UMLS and MetaMap helps increase results for both PH (from 68.3% to 74.9%) and DS (from 61.4% to 74.3%).

**Figure 8:**
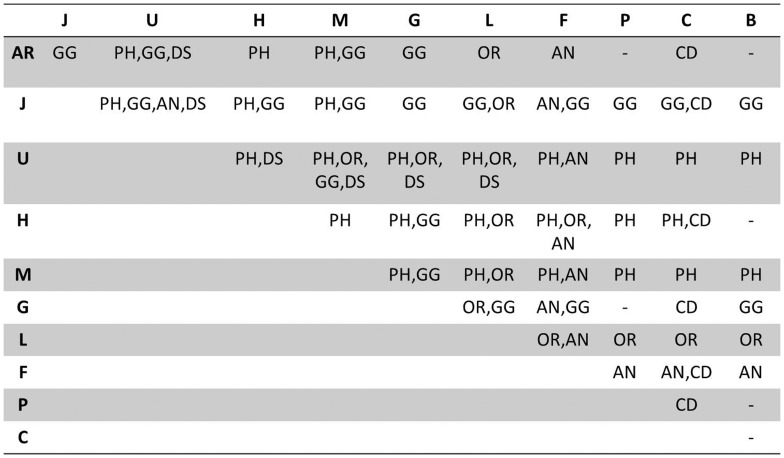
Statistical significance tests for differences in performance using approximate randomization on resources contributions. The entries in cells indicate that the two systems are significantly different in F-scores. AR: All resources, J: JNLPBA model, U: UMLS and MetaMap, H:Human Phenotype Ontology, M: Mammalian Phenotype Ontology, G: Gene Dictionary from NCBI, L: Linnaeus, F: Foundation Model of Anatomy, P: Phenotypic Trait Ontology, C: Jochem's dictionary, B: Brenda Tissue Ontology, -: No significant difference. Significance is decided at p< = 0.05.

Using the approximate randomization approach we calculated significance scores for these results. These are shown in Figure 8 and highlight resource contributions with the rows and columns showing which resource was not used in the system (e.g. *J* means the system did not use JNLPBA trained ME+BS model feature, *AR* means all resources are used). The corresponding cell shows entities which have a significance test value for difference in performance between two systems with p< = 0.05. For example, the cell in row *AR* and column *H* marked with *PH*, means there was a significant test value for *PH* for difference in performance when a system without HPO (H) was compared to a system with All Resources (AR) with p< = 0.05. Hyphen (*-*) stands for No significant difference', meaning that there is no entity which has significant test value with p< = 0.05. The significance scores highlight the contribution of UMLS to three NE classes (BG,GG and DS), the MP to phenotype candidates (PH) and GG, as well as the ineffectiveness of PATO for our corpus.

#### Resolution methods

In the resolution module we used three separate method for resolving conflict: a rule-based method (priority list), Maximum Entropy with beam search decoding and SVM learn-to-rank. The results are shown on Table 8. Maximum Entropy has the worst results with F-score of 74.9. F-score for the Priority list approach is 79.2% and SVM learn-to-rank has the best result with 79.9%. SVM learn-to-rank shows its advantage compared to the Priority List approach across almost all entity classes, included PH, GG, CD and AN with the exception of OR and DS. Table 9 shows the significance test results for the resolution module.

**Table 8:** Performance of named entity recognition using Priority List (PL), ME plus beam search (ME+BS)and SVM learn-to-rank (SVM+LTR).

	PL			*ME+BS*			*SVM+LTR*		
NE class	P	R	F	P	R	F	P	R	F
PH	73.7	76.1	74.9	73.3	68.2	70.7	74.3	76.4	**75.3**
GG	87.0	83.5	85.2	84.7	84.0	84.4	86.8	85.0	**85.9**
OR	72.8	78.1	**75.4**	62.1	65.9	63.9	70.2	77.2	73.5
CD	79.6	81.3	80.4	74.2	71.6	72.9	80.5	81.4	**80.9**
AN	72.4	82.5	77.1	69.4	71.6	70.5	75.6	80.1	**77.8**
DS	75.8	72.9	**74.3**	71.9	70.4	71.1	73.2	71.6	72.4
ALL	-	-	79.2	-	-	74.9	-	-	**79.9**

Each horizontal row shows Precision, Recall and F-score performance for a class using alternative methods. *ALL* shows micro-averaged F-score.

**Table 9:** Statistical significance tests for differences in performance using approximate randomization on Resolution methods.

	Priority list	ME+BS
SVM LRT	GG, OR	PH, GG, OR, AN, DS
Priority list		PH, GG, OR, AN, DS

The entries in cells indicate that the two systems are significantly different in F-scores. CD has no significant difference for all tests. Significance is decided at p< = 0.05.

Because the difference between results of SVM learn-to-rank and Priority List is quite small (0.7%), we try to investigate the results in more detail in the Discussion section below to get an understanding behind the complex contributing factors.

In order to obtain and understanding about how the model performed on unique mentions, i.e. those that did not appear in the training set, we provide a side by side comparison in Table 10. The table shows a relatively large fall in performance for phenotypes from 75.3% to 62.8%. The drop in performance for each class appears proportional to the rate of unique entities.

**Table 10:** Performance of named entity recognition using SVM learn-to-rank (SVM+LTR) for all entities in the cross-validation test and unique entities only.

	*All mentions*			*Unique only*			Unique
NE class	P	R	F	P	R	F	Rate
PH	74.3	76.4	**75.3**	65.4	60.3	62.3	26.2
GG	86.8	85.0	**85.9**	80.2	79.4	79.8	14.6
OR	70.2	77.2	73.5	67.3	69.3	68.3	22.9
CD	80.5	81.4	**80.9**	74.3	71.0	72.6	41.3
AN	75.6	80.1	**77.8**	71.3	72.6	72.0	19.2
DS	73.2	71.6	72.4	70.1	69.2	69.7	12.3
ALL	-	-	**79.9**	-	-	73.2	-

Each horizontal row shows Precision, Recall and F-score performance for a class using alternative methods. *Unique Rate* shows the percentage of unique entity mentions seen in the cross-validation test for each class. *ALL* shows micro-averaged F-score.

### Discussion

Our first impression was that the use of all resources had contributed to increasing the results. Examples of mentions in the corpus where we noticed a gain in recall with each of the resources are given in Table 11.

**Table 11:** Examples of mentions in the corpus where we noticed a gain in recall with each of the resources.

No.	Resource	Entity example	Named entity class
1	JNLPBA ME+BS	[human gammaglobulin]	PH
	corpus	[eukaryotic elongation factor 1A-1]	PH
		[high-affinity human mAb]	PH
2	UMLS &	[disorder of the Steroidogenic Acute	PH
	MetaMap	Regulatory Protein]	
		[Dermatitis Herpetiformis]	DS
		[uveitis]	DS
3	HPO	[immunoglobulin abnormality]	PH
		[asthma phenotype]	PH
		[autoimmunity]	PH
4	MP	[oxidative stress pathway]	PH
		[intestinal inflammation]	PH
		[insulitis]	PH
5	Gene	[CEACAM6]	GG
	dictionary	[COL29A1]	GG
		[Slc30A8]	GG
6	Linnaeus	[adenoviruses]	OR
		[murine]	OR
		[adherent-invasive E. coli]	OR
7	FMA Ontology	[lung]	AN
		[multiple organ systems]	AN
		[central nervous system]	AN
8	PATO	[high IgE levels]	PH
9	Jochem	[S €nitrosoglutathione]	CD
	dictionary	[histamine]	CD
		[dapsone]	CD
10	Brenda Tissue	[ileal mucosa]	AN
	ontology		

Named entity class is the correct results.

The greatest contributions we observed came from Jochem's dictionary for CD (+38.8%) and Linnaeus for OR (+25.5%). We interpret this result as reasonable because of the referential semantics and scoping of our entity mentions as well as the completeness of these resources: OR contains many generic references which are very hard to recognize for the machine learning labeler or the rule-based labeler (such as [family], [case], [cohort], etc.), Linnaeus helped to resolve these cases; Jochem's dictionary is a very large and comprehensive resource which combines information from UMLS, MeSH, ChEBI, DrugBank, KEGG, HMDB, and ChemIDplus.

Both HPO and MP affect PH's results in a positive way. However although the two resources both look at phenotypes, what they contribute is quite different because of their structures. Note that we estimated the overlap between HPO and MP using approximate string matching giving an estimate for overlap of about 481 root terms, or 4.9% of the HPO root terms and 5.5% of the MP root terms. The phenotype mentions in our corpus appear to be more similar to MP than HPO (MP increase PH's results by +20.5% while HP increased PH's results by +13.1%). It is worth noting that some PH mentions are not recognisable directly in either resource although with transformation and the application of semantic functions such as generality matching this should improve. For example, *serum total immunoglobin* as a PH would match to the MP entry *abnormal serum level of immunoglobin/increased serum level of immunoglobin G*. To avoid an unacceptable increase in false negatives this requires deeper semantic analysis than we have provided here, to decompose the term into entity and quality parts. We will focus more on this in future work.

With regard to anatomical entities it is clear that the FMA has greater coverage on the Phenominer A corpus than the Brenda Tissue Ontology which focuses on tissue. This results in the FMA gaining AN +18.1% whereas using the Brenda Tissue Ontology only gave +1.1%. For genes and proteins, using a sequence labeler trained on the JNLPBA corpus resulted in GG's result increasing by +14.2% but using the NCBI Gene Dictionary only gave an increase of +2.5%.

Finally, the UMLS and MetaMap have been shown to be effective cross-class resources, using them increased results for both PH by +6.6% and DS by +12.9%.

In Table 12, we show several examples of errors by the Priority List and SVM learn-to-rank. Examples 1 and 2 show where the Priority List disagreed with the gold standard annotation about a mistaken disease mention but SVM learn-to-rank agreed. In example 3, the Priority List is correct but SVM learn-to-rank is incorrect.

**Table 12:** Errors by resolution module using Priority List (PL) and SVM learn-to-rank (SVM LTR).

No.	Entity	CA	ML	RB	DB					*Merge module*	
					PH	GG	DS	CD	AN	PL	LTR
1	[susceptibilities to	PH	PH	-	-	-	DS^a^	-	-	DS	**PH**
	autoimmune disease]										
2	[asthma and	PH	PH	-	PH^b^	-	DS^c^	-	-	DS	**PH**
	atopy phenotypes]										
3	[IgE levels]	PH	GG	-	PH^d^	-	-	-	-	**PH**	GG
4	[Toll-like receptor/	PH	GG	-	-	GG^e^	-	-	-	GG	GG
	IL-1R pathways]										
5	[MyD88-deficiency]	PH	GG	-	-	-	-	-	-	GG	GG
6	[allergen-induced	PH	DS	-	-	-	-	-	-	DS	DS
	bronchial										
	inflammation]										

CA: Corpus annotation. Key to labeler: ML: Machine Learning labeler, RB: Rule-based labeler, DB: Dictionary-based labeler. PL: Priority list, LTR: SVM- Learn to rank. The resources which the dictionary-based labelers used to recognize the entity are as follows:

*^a^*UMLS C0004364,

*^b^*HP 0002099,

*^c^*UMLS C0004096,

*^d^*MP 0002492 and HP 0003212,

*^e^*NCBI Gene dictionary.

The Priority List method appears in a minority of cases to be too strict where there is ambiguity in making a choice. These include systematic ambiguities between DS and PH, OR and DS, PH and OR, etc. For example, the Priority List gives a higher priority to DS over PH. This rule is correct in the case of diseases included in the HPO (e.g. *[asthma]_DS_, [allergy]_DS_*) but it is incorrect if entities have the form: phenotype of disease' (e.g. *[addison disease only (ADO) phenotype]_PH_, [asthma-related phenotypes]_PH_, [pathogenesis of early-onset persistent asthma]_PH_*). Similarly, the rule giving DS priority over OR is correct if a disease appears in human or mouse (*[human autoimmune disease]_DS_*) but incorrect if a particular individual has a disease (e.g. *[lupus patients]_OR_, [non-obese diabetic (NOD) mouse]_OR_*). For these ambiguities, SVM learn-to-rank shows its advantage, as it is more flexible than the Priority List and can choose the final label based on many factors.

However, in many cases the Priority List is still a strong choice of resolution method. For example, based on our ontological analysis of PH and GG it is often possible for a GG to form a fully embedded part of a PH mention. Non-conforming examples seem to be very rare. Thus, the rule that PH takes priority over GG may bring correct results in the majority of cases while SVM learn-to-rank's flexibility is unneeded.

Finally, it is important to mention that our resolution module only affects the final output if ambiguity is detected. Rows 4–6 in Table 12 show examples of where both the Priority List and SVM learn-to-rank disagreed with the Phenominer A annotation. Because there isn't any labeler output conflict, the incorrect final results come from the incorrect results of input modules.

## Conclusions

In this article we have presented a systematic study of how to combine sequence labels from various ontological resources and methods in an attempt to address the task of phenotype candidate recognition. The study is the first we believe to evaluate such a rich set of features for the complex class of phenotypes. Our system achieved the best micro-averaged F-score for the six entity classes of 79.93 with 75.31 for phenotype candidates in the auto-immune domain. We observed the advantage of using SVM learn-to-rank for hypothesis resolution and using all resources. We conclude that selected semantic types such as chemicals and genes are well covered by single semantic resources whereas phenotype candidates require combinations. In this respect key roles were observed for the Mammalian Phenotype Ontology, the Human Phenotype Ontology and the UMLS.

Our approach has coped well with the compositional structure of phenotype representations. We note though that so far we have used these ontologies as terminology resources and there will undoubtedly be potential to exploit the structures within their hierarchies in ways that can extend performance further. Beyond this, the next step is to take the phenotype candidates and decompose them according to domain concepts, i.e. to ground them. This will allow free text articles to be linked through community vocabularies, streamlining phenotype vocabulary and enabling the systematic investigation of disease-gene relationships through textual data integration.

## Supporting Information

Data S1 **Annotated data for the auto-immune corpus of PubMed abstracts.**
(ZIP)
